# Supplementary material for: Content-rich biological network constructed by mining PubMed abstracts
Source: BMC Bioinformatics. 2004 Oct 8;5:147. doi: 10.1186/1471-2105-5-147 (PMC528731; doi:10.1186/1471-2105-5-147)
Supplement: Additional File 2 — The original results of the above study (non-essential files are deleted to keep the file size under the limit set by BMC bioinformatics). [file 1471-2105-5-147-S2.bz2 › chilibotAdditionalFile2/dip05/737ID9501520E18103/html/EVGA_EVGS.html]

 


 **EVGA** and **EVGS** 
  
Found 7 abstracts in PubMed, retrieved 05.  
 

 What does Google say? 
 PDF only 
| .edu only 

---

**Interactive relationship** (e.g. stimulation, inhibition, etc)

**Neutral relationship**- No interaction was observed either between  **EvgA**  and Bordetella BvgS or BvgA and  **EvgS** .  Ref: 9501520 Biosci Biotechnol Biochem, 1998
- In agreement with these data, surface plasmon resonance studies revealed a 2 1 stoichiometry in the interaction of  **EvgA**  with the immobilized  **EvgS**  HPt domain and an affinity constant of 1.  Ref: 10825546 Biochim Biophys Acta, 2000
- In contrast, the hybrid protein consisting of the BvgS transmitter and the  **EvgS**  receiver and HPt domains BvgS T  **EvgS**  RO was unable to phosphorylate BvgA but efficiently phosphorylated  **EvgA** .  Ref: 9535079 Mol Microbiol, 1998
- Under equimolar conditions, significant in vitro phosphorylation of purified BvgA and  **EvgA**  proteins was only obtained by their histidine kinases, BvgS and  **EvgS**  respectively.  Ref: 9535079 Mol Microbiol, 1998
- These mutations were found to be single nucleotide substitutions within the linker region of the sensor protein  **EvgS**   [ **EVGS** ] , which is part of a two component regulatory system along with  **EvgA** .  Ref: 10923791 Biosci Biotechnol Biochem, 2000
- In the case of the response regulators, the receiver but not the output domains contribute to the specific interaction with the histidine kinases, because a hybrid protein consisting of the  **EvgA**  receiver and the BvgA output domain could only be phosphorylated by the  **EvgS**  protein.  Ref: 9535079 Mol Microbiol, 1998

**Non-interactive relationship** (e.g. studied together, co-existance, homology, etc.)

- One hybrid histidine kinase consisting of the BvgS transmitter and HPt domains and of the  **EvgS**  receiver domain BvgS TO  **EvgS**  R was able to phosphorylate BvgA but not  **EvgA** .  Ref: 9535079 Mol Microbiol, 1998
- **EvgA**  and  **EvgS**  constitute one two component signal transduction system in Escherichia coli.  Ref: 9501520 Biosci Biotechnol Biochem, 1998
- Oligomerization of the effector proteins  **EvgA**  and BvgA and of truncated  **EvgS**  and BvgS derived signalling proteins containing the receiver and histidine containing phosphotransfer HPt domains or comprising only the HPt domains were characterized by native gel electrophoresis, gel permeation experiments and analytical ultracentrifugation.  Ref: 10825546 Biochim Biophys Acta, 2000
- Here, we investigated protein to protein interactions between  **EvgA**  and  **EvgS**  and also between the EvgAS system and other related signaling pathways by means of surface plasmon resonance.  Ref: 9501520 Biosci Biotechnol Biochem, 1998
- Transcription of emrKY is regulated by the  **EvgA**   **EvgS**  two component system in Escherichia coli K 12.  Ref: 10923791 Biosci Biotechnol Biochem, 2000
- OmpR, a response regulator for the osmoregulative gene expression of E. coli, had similar but not identical behavior towards  **EvgS**  to that of  **EvgA** .  Ref: 9501520 Biosci Biotechnol Biochem, 1998
- **EvgA**  and  **EvgS**  interacted directly and inhibition of phosphorylation of their functional domains abolished formation of the EvgAS complex.  Ref: 9501520 Biosci Biotechnol Biochem, 1998
- The evgAS operon of Escherichia coli encodes the  **EvgA**  response regulator and the  **EvgS**  sensory kinase, which are members of one of the two component signal transduction systems of Escherichia coli.  Ref: 9532784 Biosci Biotechnol Biochem, 1998
